# Physical Education and the Adoption of Habits Related to the Mediterranean Diet

**DOI:** 10.3390/nu13020567

**Published:** 2021-02-09

**Authors:** María-Jesús Lirola, Rubén Trigueros, Jose M. Aguilar-Parra, Isabel Mercader, Juan M. Fernandez Campoy, Mª del Pilar Díaz-López

**Affiliations:** 1 Department of Physical Education, University of Almería, 04120 Almería, Spain; mariajesus.lirola@ual.es; 2 Hum-878 Research Team, Health Research Centre, Department of Psychology, University of Almería, 04120 Almería, Spain; 3 Department of Education, University of Almería, 04120 Almería, Spain; jfc105@ual.es; 4 Hum-498 Research Team, Health Research Centre, Department of Nursing Science, Physiotherapy and Medicine, University of Almería, 04120 Almería, Spain; dlm477@ual.es

**Keywords:** child obesity, motivation, physical education, healthy habits

## Abstract

Childhood obesity and sedentary lifestyles are now gaining a foothold in the Western world. The aim of this research was to analyse the influence of physical education classes on a healthy diet (i.e., Mediterranean diet). To this end, psychological constructs derived from the theory of self-determination and the theory of planned behaviour were taken into account, such as the satisfaction and frustration of basic psychological needs, motivation in physical education classes, and social cognition and intention. A total of 3415 secondary school students (13–19 years) participated in this study. A structural equation model was proposed that would explain the relationships between the variables mentioned above and the adherence to a Mediterranean diet. The results provide adequate fit indexes for the proposed model. Based on the results of the study, it was concluded that a high satisfaction perceived in the physical education classes would help to reinforce the intention of having a healthy diet and therefore help to generate a perdurable commitment to this habit.

## 1. Introduction

Today, the number of people suffering from obesity has reached alarming rates. According to the World Health Organization [1], the data can be considered epidemiological. In Western countries where the socio-economic level is high, it is known that the rate of childhood obesity has been increasing in recent years [2,3]. Moreover, recent research has shown that the recent confinement of the general population to their homes, as a result of the pandemic caused by the coronavirus (COVID-19), has contributed toward many people living a more sedentary life than before [4]. The consequences of sedentary lifestyles and poor eating habits can be found at various levels of health. At the physical level, we find high probabilities of suffering diseases (i.e., metabolic, cardiovascular, immune system depression, and even certain types of cancer), which are related to a high mortality rate [5]. On a psychological level, sedentary lifestyles and poor eating habits are associated with low self-esteem and high body dissatisfaction [6]. On a social level, it is known that adolescents with high body mass indexes are more likely to suffer from bullying [7].

The current situation needs to be improved. In this sense, research has shown that the acquisition and maintenance of correct healthy habits are developed and incorporated into people’s lives from childhood [8]. Therefore, it is very useful to work on healthy habits and contents related to a balanced diet from an early age, thus extending the benefits to adulthood.

The child and youth population invests an important part of its time in educational institutions. In particular, children from 3 to 16 years of age must attend their respective schools regularly and compulsorily. Students in infant and primary education spend a total of 25 h of classes per week in educational facilities [9], and for those who are enrolled in secondary education, the number of hours they spend in classes is stipulated at 30 h of classes per week [10]. Therefore, because we invest a significant amount of time in educational institutions during the years of childhood and early youth, the study of their influence and impact on the development of correct healthy habits proves to be of interest.

In this sense, physical education (PE) classes have an important role in putting children in the right direction and providing them with the necessary motivation to adhere to healthy habits (i.e., an active life and balanced diet). In recent decades, one of the best known theories on the study of human motivation is self-determination theory (SDT) [11], which, in one of its postulates, underlines the importance of the influence of the educational context on the behaviour and attitudes of its students. Thus, satisfaction with classes has been widely studied due to, among other factors, the possibility of developing healthy habits that remain in adulthood [12]. Therefore, in order to know the degree of satisfaction with PE lessons, attention must be paid to the distribution of the sessions, how the children are organised, the feedback given by the teacher, and the progress of the proposed activities, among others. Because the context of PE can be complex to measure, different investigations have developed and successfully validated a questionnaire made to evaluate the level of satisfaction of students in PE classes [13,14,15]. In this sense, three dimensions are highlighted as variables to be considered: (a) teaching, related to the perception of the teacher’s attitude and explanations; (b) cognitive development, related to the perception of improvement in cognitive performance; and (c) mastery experiences, related to the perception of improvement in physical performance.

The relation between perceived satisfaction in PE classes and the development of adaptive behaviours in students, such as participation in physical activities, and their long-term effects has been studied by several researchers [16,17]. To this extent, satisfaction would also lead to the satisfaction of basic psychological needs (BPNs). According to the SDT, human beings’ behaviours are regulated by three basic psychological needs; when these needs are satisfied, it leads to well-being and personal development, but when they are thwarted, it may lead to discomfort and maladaptive behaviours [18]. BPNs are composed of the need for competence, autonomy, and relationships. Competence is related to the ability a person has when it comes to carrying out a specific task; autonomy refers to the capacity to be independent and have control over the choices we make; and a relationship is that which arises from the connection with others and is associated with the feeling of creating bonds with other people [19]. The satisfaction of all three BPNs results in the regulation of behaviour, which may be determined by intrinsic or extrinsic motivation. Intrinsic motivations promote activity performance by personal motivations that are not related to external elements or people; extrinsic motivation is determined by external factors [20]. Based on the SDT, it is known that more self-determined behaviours lead to better and more adherent behaviour over time towards the activity or activities that cause this type of motivation and that less self-determined behaviours are related to premature abandonment and low adherence and commitment to the activity or activities involved [21].

Based on the theory of planned behaviour (TPB), it is known that behaviour is also determined by behavioural intent [22,23]. This intention can be explained by three previous factors called social cognition: (a) the subjective norm, which refers to the perception that certain behaviours are common and/or considered appropriate in one’s social environment; (b) the attitude towards the behaviour, which would be the evaluation that an individual makes about the behaviour; and (c) the perceived behavioural control (PBC), which is related to the capacity or ability that a person perceives that they may have to carry out a behaviour. In this way, the intention to carry out a certain activity would come into play in the study of possible psychological factors that could offer a greater explanation in the creation and maintenance of healthy behaviours. In this sense, it becomes easier to study and/or predict the performance of a future behaviour that can be maintained over time. Therefore, the focus of this research is on the constructs that are involved in the development of healthy habits (i.e., adherence to a healthy diet), as the Mediterranean diet is considered to be rich in fibre and vegetables and to have a balance between all the recommended macronutrients [24].

Following the postulates of SDT and TPB, the present study aimed at analysing the perceived satisfaction of students in physical education classes in relation to BPNs, the level of motivation of adolescents, and behavioural intention as determining factors of behaviours related to eating habits. For this purpose, the following hypotheses were proposed: (a) teaching, mastery experiences, and cognitive development will positively predict the satisfaction of BPNs and negatively predict the thwarting of BPNs; (b) the satisfaction of BPNs will positively predict self-determined motivation; (c) the thwarting of BPNs will negatively predict self-determined motivation; (d) self-determined motivation will positively predict all three dimensions of social cognition; (e) social cognition will positively predict behavioural intention; and (f) behavioural intention will positively predict the Mediterranean diet.

## 2. Method

### 2.1. Participants

From different secondary schools in the provinces of Malaga and Almeria (Spain), 3415 (1659 male and 1756 female) students aged 13–19 participated (M = 15.31; SD = 1.68). The sampling method was non-probabilistically incidental.

### 2.2. Measurement

#### 2.2.1. Teaching Environment

The validated and adapted version of the Physical Activity Class Satisfaction Questionnaire (PACSQ) [15] was used in the Spanish context of PE by Sicilia et al. [13]. The questionnaire is headed by a sentence referring to the students’ satisfaction towards PE classes. The questionnaire has 45 items that are distributed into nine factors. However, only the three following factors of the scale were used: cognitive development, mastery experiences, and teaching. The answers given by the students were on a Likert-type scale (strongly disagree (1) to strongly agree (8)).

#### 2.2.2. Satisfaction of Basic Psychological Needs

The version adapted and validated to the Spanish context [25] from Basic Psychological Needs in Physical Education (BPN-PE; [26,27,28]) was used. The questionnaire has 18 items that are distributed into four factors (competence, autonomy, novelty, and relatedness). The answers given by the students were on a Likert-type scale (totally disagree (1) to totally agree (7)).

Frustration of basic psychological needs: The version adapted and validated to the Spanish context [29] from Psychological Needs Thwarting Scale in Physical Exercise [30] was used. The questionnaire has 17 items that are distributed into four factors (competence, autonomy, novelty, and relatedness). The answers given by the students were on a Likert-type scale (not true at all (1) to completely true (7)).

#### 2.2.3. Motivation

The version adapted and validated to the Spanish context [31] from Academic Self-Regulation Scale [32] was used. The questionnaire has 24 items that are distributed into six factors (intrinsic motivation, integrated regulation, identified regulation, introjected regulation, external regulation, and amotivation). The answers given by the students were on a Likert-type scale (not true (1) to completely true (7)).

For the present study, we chose to calculate the self-determination index (see, [33]). This index has been used in numerous studies and has been determined to be valid and reliable.

Social Cognition and Intention: Four factors were used: subjective norm, intention, perceived behavioural control, and attitude from the theory of planned behaviour. These scales have been successfully used in several studies [34,35] referring to healthy and balanced eating. The answers given by the students were on a Likert-type scale (strongly disagree (1) to strongly agree (7)), except for one item of the subjective norms factor (no control (1) to strong control (7)).

Balanced Diet: The Spanish version of the scale linked to the Mediterranean diet was used [36]. This scale consists of 16 items, with the overall score ranging from 0 to 12.

### 2.3. Procedure

At the beginning of the study, schools were asked to cooperate in order to gain access to the students. Once in the classroom, the students received an explanation the objectives of the study and were given an informed consent to be signed by their parents/legal guardians, as they were minors.

The completion of the questionnaires by the students was done at the beginning of the PE classes, anonymously. A staff member from the project group was in place to assist in answering any questions that arose. The students completed the questionnaires in about 30 min.

This study respected the tenets of the Declaration of Helsinki. Furthermore, and it was approved by the bioethics committee from University of Almeria (Ref. UALBIO 2019/014).

### 2.4. Data Analysis

Using the statistical programme SPSS v.25 (IBM, Armonk, NY, USA), descriptive statistical analyses and reliability analyses were conducted using Cronbach’s alpha. Using the AMOS v.20 (IBM, Armonk, NY, USA) statistical programme, structural equation modelling analyses were conducted. For this analysis, the maximum likelihood method was applied together with 7000 interactions in bootstrapping.

Table 1 shows the different fit indices for accepting or rejecting the model [37,38].

It should be noted that these indexes should be treated with precaution, due to them being considered excessively strict or difficult to reach in large models [39].

## 3. Results

### 3.1. Preliminary Analysis

As shown in Table 2, the bivariate correlations between the factors in the study were positive. The exception was with correlations related to frustration of BPNs. As for the reliability analysis, the scores were above 0.70 [40] and were therefore considered to be reliable.

### 3.2. Structural Equation Modelling

**Figure 1:**
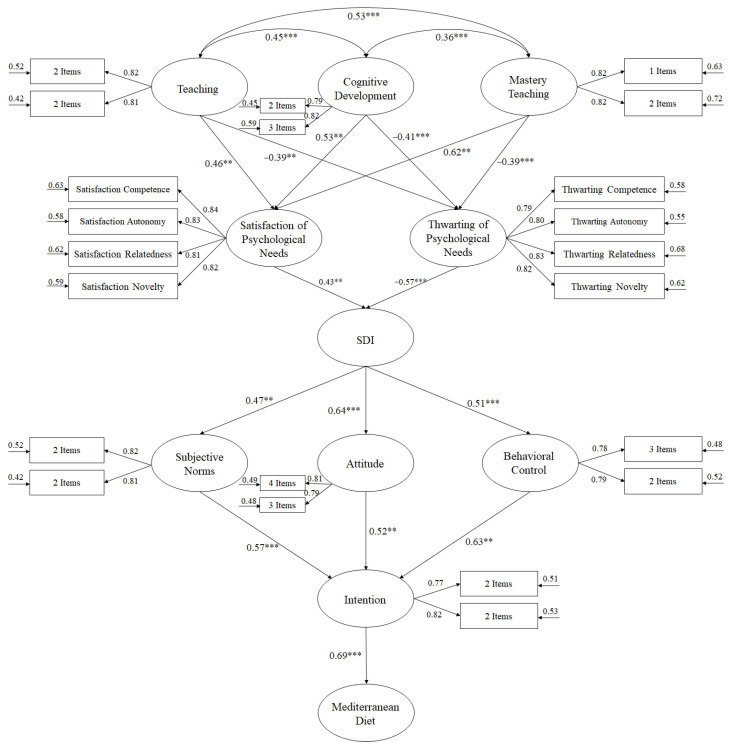
Predictive model. Structural equation model. Note: *** *p* < 0.001; ** *p* < 0.01.

Using a SEM (Figure 1), the predictive relationships between the factors were analysed, resulting in appropriate adjustment rates: χ^2^ (236, *N* = 3415) = 652.65, χ^2^/*df* = 2.48, *p* < 0.001, Incremental Fit Index (IFI) = 0.96, Comparative Fit Index (CFI) = 0.96, Tucker Lewis Index (TLI) = 0.96, Standardized Root Mean Square Residual (SRMR) = 0.048, and Root Mean Square Error of Approximation (RMSEA) = 0.057 (IC90% = 0.053–0.064). The correlations between the study variables were analysed using standardised regression weights.

## 4. Discussion

The aim of this study was to analyse the perceived satisfaction of students in physical education classes in relation to basic psychological needs, the level of self-determination of adolescents, and behavioural intention. All of the above factors were investigated as possible determining factors when predicting adherence to a Mediterranean diet.

Firstly, descriptive statistics, the reliability of the instruments used, and the bivariate correlations between the constructs that were studied were calculated. The results showed values that supported the reliability of the scales used. The correlations were significant in their entirety and positive, except for those related to the frustration of basic psychological needs, which showed a negative sign. These results are in line with previous studies where the factors composing PE class satisfaction, BNPs satisfaction, self-determined motivation, factors composing social cognition, behavioural intention, and following a Mediterranean diet were determined to be constructs positively related to each other because of their positive connotations [41,42,43] and studies that have demonstrated that BPNs thwarting is negatively related to all of them [44,45].

Secondly, a structural equation model was developed. The results derived from this analysis show how the three dimensions used in the PE class satisfaction questionnaire positively and significantly predicted the satisfaction of the BPNs and negatively and significantly predicted the frustration of the BPNs, as was observed in previous research [46]. Moving forward in the model, the self-determination index was significantly and positively predicted by the satisfaction of the BPNs and significantly and negatively predicted by the frustration of the BPNs; this fact is reinforced by the multiple previous studies carried out in the framework of the SDT, where such relationships are found [47,48,49].

Continuing with the central part of the model, constructs belonging to two different theories are related: on the one hand, the self-determination level from the SDT and, on the other hand, the three factors that conform to the construct of social cognition and that have their origin in the TPB. The level of self-determination positively and significantly predicted the factors of social cognition. These results are in line with the central principle of the models that integrate SDT and TPB, which shows how people strategically align their beliefs with their self-determined motives in order to carry out certain activities or behaviours that satisfy their needs in the future, as Hagger and Chatzisarantis [50] previously found for the case of physical activity. Recently, it has been possible to confirm, through a longitudinal study, that the sense of prediction places self-determined motivation as a temporal antecedent of the factors of social cognition, showing greater effects in this sense if compared to its opposite distribution [51]. On the other hand, factors shaping social cognition significantly and positively predicted the intention to follow a healthy diet, which reinforces previous findings [52,53].

Finally, the last part of the model shows how behavioural intention positively and significantly predicted the adherence to a Mediterranean diet, which is in line with the results obtained by Margüenda, Contreras, Bermúdez, and Pérez [54], where behavioural intention yielded values that predicted the development of healthy behaviours. Behavioural intention has been shown to be a precursor of the development of such behaviour independently of the behaviour to be assessed [55,56].

To date, although the relationships established above have been analysed in various studies, no one has been found to have studied their behaviour as part of a structural equation model. This study sheds light on the psychological processes involved in and influencing the future maintenance of healthy habits, such as, in this case, adherence to the Mediterranean diet. The limitations that are present in this research should be highlighted. Firstly, this is a cross-sectional study, and it is therefore not possible to carry out or interpret results between the proposed cause-and-effect relationships. Additionally, the sample of convenience reduces the possibility of making generic statements with a certain degree of scientific rigour. As prospective research, a longitudinal study is proposed. It would also be of great interest to extend the study to a younger sector of the population, because the development and maintenance of healthy habits over time lies in the acquisition of these habits from an early age.

## 5. Conclusions

The results found in this research are in line with the theoretical postulates of the SDT and the TPB, showing and bringing light to the study of the prediction of healthy behaviours that last over time. In this case, the importance of the perception of satisfaction with PE classes for the adolescents participating in this study was highlighted in relation to presenting an adherence to the Mediterranean diet.

## Figures and Tables

**Table 1:** Adjustment indexes.

Statistics	Good Indexes
χ^2^/degree freedom	Between 2 and 3
Comparative Fit Index (CFI)	+0.95
Incremental Fit Index (IFI)	+0.95
Tucker Lewis Index (TLI)	+0.95
RMSEA (Root Mean Square Error of Approximation) CI 90%	Equal or less than 0.06
SRMR (Standardized Root Mean Square Residual)	Equal or less than 0.08

**Table 2:** Descriptive statistics, reliability, and correlations between all variables.

Factors	M	SD	α	1	2	3	4	5	6	7	8	9	10	11
1. Cognitive Development	5.48	1.12	0.83	-	0.42 **	0.45 **	0.40 ***	−0.35 **	0.59 ***	0.61 ***	0.27 **	0.65 ***	0.68 ***	0.52 ***
2. Mastery Experiences	6.21	1.53	0.84		-	0.28 **	0.51 ***	−0.42 **	0.52 **	0.66 **	0.39 **	0.38 **	0.59 ***	0.39 ***
3. Teaching	6.42	1.43	0.88			-	0.61 **	−0.36 **	0.48 ***	0.54 ***	0.46 ***	0.42 **	0.61 ***	0.38 ***
4. BPNs Satisfaction	5.98	1.46	0.83				-	−0.58 ***	0.47 **	0.34 **	0.37 **	0.47 ***	0.48 ***	0.42 **
5. BPNs Thwarting	2.16	1.69	0.82					-	−0.31 **	−0.36 ***	−0.41 ***	−0.46 **	−0.37 **	−0.13 *
6. SDI-PE	15.37	8.46	-						-	0.46 ***	0.39 **	0.57 ***	0.78 ***	0.47 **
7. Subjective Standards	4.88	1.36	0.79							-	0.51 ***	0.61 ***	0.69 **	0.49 ***
8. Behavioural Control	4.35	0.99	0.77								-	0.5 **	0.63 **	0.57 **
9. Attitude	4.66	1.36	0.80									-	0.52 ***	0.43 ***
10. Intention	5.13	0.98	0.82										-	0.49 **
11. Mediterranean Diet	5.99	1.88	0.85											-

*** *p* < 0.001; ** *p* < 0.01; * *p* < 0.05. Note: BPNs: basic psychological needs; SDI-PE: Self-Determination Index in Physical Education; PE: physical education.

## Data Availability

Not applicable.
